# Bariatric Surgery and Myocardial Mechanics: A Meta-Analysis of Speckle Tracking Echocardiographic Studies

**DOI:** 10.3390/jcm11164655

**Published:** 2022-08-09

**Authors:** Elisa Gherbesi, Cesare Cuspidi, Andrea Faggiano, Carla Sala, Stefano Carugo, Marijana Tadic

**Affiliations:** 1 Department of Clinical Sciences and Community Health, University of Milano and Fondazione Ospedale Maggiore IRCCS Policlinico di Milano, 20122 Milan, Italy; 2 Department of Medicine and Surgery, University of Milano-Bicocca, 20126 Milan, Italy; 3 Department of Cardiology, University Clinical Hospital Centre “Dragisa Misovic”, Heroja Milana Tepica 1, 11000 Belgrade, Serbia

**Keywords:** bariatric surgery, left ventricular strain, left ventricular ejection fraction, morbid obesity

## Abstract

Aim: Current evidence on the effects of bariatric surgery on cardiac mechanics in patients with obesity is based on a few single studies. We investigated this issue through a meta-analysis of speckle tracking echocardiography (STE) studies that reported data on changes in left ventricular (LV) mechanics as assessed by global longitudinal strain (GLS). Methods: The PubMed, OVID-MEDLINE and Cochrane library databases were systematically analysed to search English-language articles published from inception to 31 May 2022. Studies were identified by using Me-SH terms and crossing the following terms: “obesity”, “bariatric surgery”, “left ventricular mechanics”, “left ventricular hypertrophy”, “systolic dysfunction”, “global longitudinal strain”, “echocardiography” and “STE echocardiography”. Results: The meta-analysis, including a total of 512 patients with obesity from 13 studies (follow-up 1–23 months), showed a significant GLS improvement after bariatric procedures, with standard mean difference (SMD) being 0.50 ± 0.08, CI: 0.34/0.65, *p* < 0.0001. Corresponding SMD value for LV ejection fraction (LVEF) was 0.15 ± 0.09, CI: −0.04/0.34, *p* = 0.11. A sensitivity analysis restricted to 11 studies with follow-up ≥ 6 months confirmed that GLS (SMD: 0.47 ± 0.08, CI: 0.30/0.63, *p* < 0.0001) but not LVEF (SMD: 0.14 ± 0.11, CI: −0.08/0.37, *p* = 0.21) improved after surgery. Conclusions: Our meta-analysis adds a new piece of information on the beneficial effects of bariatric surgery on LV systolic function and, more importantly, suggests that the assessment of myocardial strain should be routinely implemented for a comprehensive evaluation of cardiac functional changes associated with bariatric procedures.

## 1. Introduction

Epidemic obesity is a growing burden for healthcare systems in both developed and underdeveloped countries, and its prevalence has dramatically increased in the last decades [1]. Overweight and obesity increase the risk for virtually all cardiovascular (CV) risk factors such as hypertension, type 2 diabetes, dyslipidaemia, metabolic syndrome and sleep disorders. Therefore, obesity is burdened with a high incidence of non-fatal and fatal CV disease, including heart failure (HF), coronary artery disease, stroke, atrial fibrillation and all-cause mortality [2,3,4]. Obesity-related changes in cardiac structure and function leading to overt CV disease have been primarily attributed for many years to hemodynamic alterations (i.e., high cardiac output pattern, volume overload); however, more recently, it has been demonstrated that neurohormonal and metabolic factors may also contribute to cardiac remodelling and abnormal ventricular function [5,6].

The fight against obesity, and its detrimental consequences, is currently based on several therapeutic options such as dietary and physical activity intervention, behavioural therapy, pharmacotherapy and bariatric surgery increasingly recommended for morbid obesity as well as in patients with less severe obesity when associated with comorbidities [7].

Compared to non-surgical interventions, bariatric surgery results in a much more marked and persistent weight loss, which in turn is associated with better modulation of CV risk factors and cardiac functions, as well as reduced all-cause and CV mortality [8,9,10]. The favourable impact of bariatric treatment on cardiac structure and function has been reported in numerous imaging studies performed since 1990. However, it is noteworthy that the meta-analyses of these studies carried out so far failed to find a consistent improvement in left ventricular (LV) systolic function, as assessed by left ventricular ejection fraction (LVEF) [11,12,13]. One of the possible explanations lies in the fact that the assessment of systolic function by LVEF has several limitations that impair its ability to identify early systolic dysfunction. More recently, novel echocardiographic techniques such as 2D and 3D speckle tracking echocardiography (STE) has gained increasing recognition as more sensitive and reliable tools in evaluating LV systolic performance, thus overcoming the inherent limitations of LVEF [14]. Starting from this background, we performed a meta-analysis of echocardiographic studies targeting the impact of bariatric surgery on LV mechanics with the aim of providing updated and comprehensive information on this relevant therapeutic issue.

## 2. Methods

### 2.1. Search and Study Selection

We reported the systematic review according to the Preferred Reporting Items for Systematic reviews and Meta-Analyses (PRISMA) guidelines [15]. Pertinent literature was systematically scrutinized to identify all papers assessing LV myocardial strain as assessed by 2D STE echocardiography in patients with obesity before and after bariatric surgery.

The PubMed, OVID-MEDLINE, and Cochrane library databases were systematically analysed to search English-language articles published from inception to 31 May 2022. Studies were identified by using Me-SH terms and crossing the following terms: “obesity”, “bariatric surgery”, “left ventricular mechanics”, “left ventricular hypertrophy”, “systolic dysfunction”, “global longitudinal strain”, “echocardiography” and “STE echocardiography” (Table S1).

Two authors (EG and AA) assessed all titles and abstracts retrieved with the search. When there was an agreement on a specific record, the full text of the study was analyzed by both reviewers to establish eligibility according to the inclusion criteria mentioned below. A third reviewer (CC) resolved disagreements on study judgments. Data extraction was performed by two reviewers (EG and AA) and independently checked by another reviewer (CC).

The main inclusion criteria were: (I) English articles published in peer-reviewed journals; (II) studies providing data on LV mechanics (i.e., GLS) by 2D STE echocardiography; (III) minimum set of clinical/demographic data; (IV) duration of follow-up longer than 1 month. Specific exclusion criteria were: (I) studies with less than 10 patients with obesity; (II) studies conducted in children and adolescents (age < 18 years); (III) reviews, editorials and case reports were excluded from analyses (but examined for potential additional references).

The Newcastle–Ottawa Scale (NOS) was used to measure study quality (http://www.ohri.ca/programs/clinical_epidemiology/oxford.htm (accessed on 1 August 2022)).

### 2.2. Echocardiographic Methods

Conventional analysis of cardiac structure and function was performed in all studies according to recommendations of contemporary guidelines. LV myocardial deformation (i.e., GLS) was measured off-line from 2D echocardiographic images using commercial dedicated software; R-R gating was used for LV strain assessment. In all studies, LV endocardium was manually traced and corrected, if necessary, and the average longitudinal strain curve was automatically provided by the software.

### 2.3. Statistical Analysis

The primary outcome of the meta-analysis was to assess the changes in LV GLS induced by bariatric surgery in obese patients. For this purpose, a pooled analysis of cardiac parameters was performed using random effects meta-analysis by Comprehensive Meta AnalysisVersion2, Biostat, Englewood, NJ. Standard means difference (SMD) with 95% confidence interval (CI) was used to calculate the statistical difference of variables of interest (i.e., LV GLS) before and after bariatric treatment.

Data provided by selected studies were expressed as absolute numbers, percentage, mean ± standard deviation (SD) and mean ± standard error (SE).

Heterogeneity was estimated by using I-square, Q and tau-square values; random effect models were applied due to the heterogeneity across studies. Meta-regression analysis was used to determine the impact of key clinical variables (i.e., body mass index changes) upon myocardial GLS. Publication bias was assessed by using the funnel plot according to the trim and fill test. Observed and adjusted values and their lower and upper limits were calculated. To assess the effect of individual studies on the pooled result, we conducted a sensitivity analysis by excluding each study one by one and recalculating the combined estimates on the remaining studies.

## 3. Results

**Figure 1:**
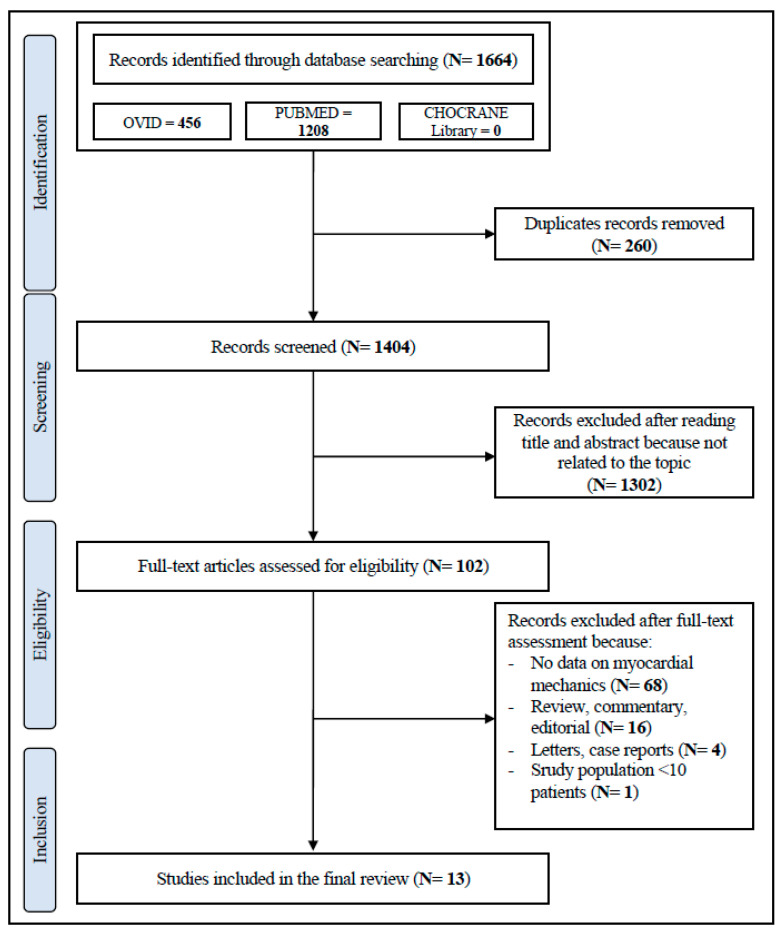
Schematic flowchart for the selection of studies.

The initial literature search identified 1404 papers. After the initial screening of titles and abstracts, 1302 studies were excluded as they were not related to the topic. Therefore, 102 studies were reviewed; of these, 77 did not report data on myocardial mechanics, and 24 were reviews, commentary, editorial articles, case reports and studies including less than 10 participants. Thus, a total of 13 studies including patients treated with bariatric surgery for a period longer than one month and containing echocardiographic data of interest were included in the final review [16,17,18,19,20,21,22,23,24,25,26,27,28] (Figure 1). According to the NOS, the quality of the studies ranged from 6 to 9 (i.e., a score that identifies studies of fair or good quality) [29]. Therefore, no study was excluded based on its limited quality (Table S2).

### 3.1. Characteristics of the Studies

On the whole 512 patients with obesity were included in 13 studies (sample size ranging from 10 to 94 participants), performed in five continental areas (Asia = 4; Europe = 4; America = 3; Africa = 1; Australia = 1).

Table 1 summarizes the main findings of selected studies such as authors, year of publication, sample size, mean age, gender, BMI and LV GLS before and after bariatric treatment, type of surgery, follow-up duration and STE method and comorbidities. Patients with previous cardiovascular events, cardiopathies and heart failure were excluded from the studies; the most frequent comorbidities were hypertension and type 2 diabetes (7 out of 13 studies).

Mean age varied from 35 ± 8 [25] to 52 ± 12 years [16]. In all studies, the prevalence of female patients was much higher than that of males. Baseline mean BMI values ranged from 40 ± 6 [19] to 51 ± 9 kg/m^2^ [15]. Corresponding values after bariatric treatment were 28 ± 4 [19] and 39 ± 4 kg/m^2^ [20]. The duration of the follow-up period varied from 1 [20] to 23 months [16]. Sleeve gastrectomy was the surgical technique used in most of the studies (8 out of 13 studies).

### 3.2. BMI and BP Changes

Baseline and post-procedural follow-up pooled mean BMI values were 45.6 ± 1.03 and 33.6 ± 1.34 kg/m^2^ (SMD: −0.54 ± 0.05, CI: −0.64/−0.45, *p* < 0.0001). Mean systolic BP decreased from 128 ± 2.7 to 120 ± 1.7 mmHg (SMD: −0.37 ± 0.07, CI: −0.52/−0.23, *p* < 0.0001); corresponding values for diastolic BP were 79 ± 2.4 and 74 ± 2.4 mmHg, respectively (SMD: −0.34 ± 0.09, CI, −0.52/−0.17, *p* < 0.0001).

### 3.3. Echocardiographic Findings

**Figure 2:**
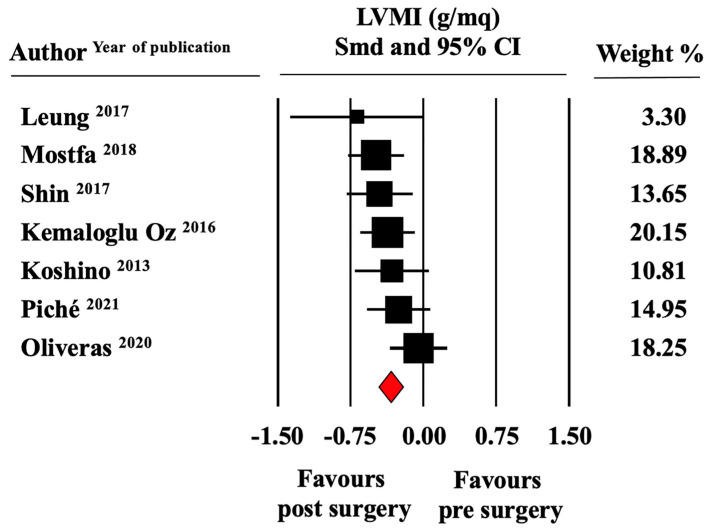
Forest plot for standard means difference (SMD) of left ventricular mass index (LVMI) in patients with obesity before and after bariatric surgery (random model). Relative weight of each study is reported on the right side. CI—confidence intervals. The filled squares represent the sample size of the study; the diamond is the average of the SMD.

Pooled left ventricular mass index (LVMI) was 119.1 ± 15.7 g/m^2^ at baseline and 100.7 ± 7.4 g/m^2^ at the end of follow-up. The meta-analysis of seven studies suggested a significant reduction of this marker of LV hypertrophy (LVH) after bariatric surgery (SMD, −0.33 ± 0.07, CI −0.46/−0.20, *p* < 0.0001) (Figure 2).

**Figure 3:**
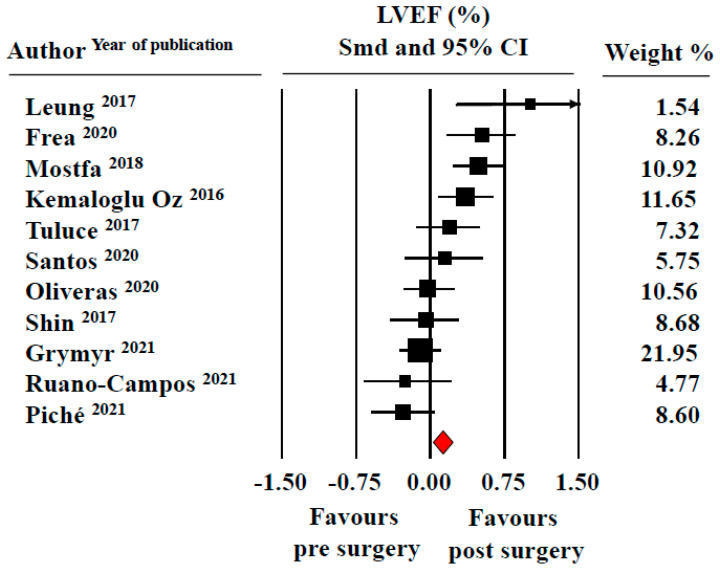
Forest plot for standard means difference (SMD) of left ventricular ejection fraction (LVEF) in patients with obesity before and after bariatric surgery (random model). Relative weight of each study is reported on the right side. CI = confidence intervals. The filled squares represent the sample size of the study; the diamond the average of the SMD.

LVEF pooled values were 61.0 ± 0.8% at baseline and 62.4 ± 1.5%at follow-up. The meta-analysis showed a non-significant increase in this index of systolic function after bariatric surgery (SMD: 0.15 ± 0.09, CI: 0.04/0.34, *p* = 0.11) (Figure 3).

The average pooled values of the ratio of early (E) peak of mitral inflow velocity to early (e) peak mitral annular velocity (E/e’ ratio), a validated LV filling pressure index, were 8.9 ± 0.4 at baseline and 8.1 ± 0.3 after follow-up (SMD, −0.31 ± 0.07, CI: −0.45/−0.17, *p* < 0.0001). The opposite trend was observed for the ratio of early (E) to late (A) peak of mitral inflow velocity (E/A ratio) being 1.23 ± 0.02 at baseline and 1.42 ± 0.09 after bariatric treatment (SMD, 0.27 ± 0.09, CI: 0.10/0.45, *p* < 0.002).

As for left atrial volume indexed to BSA (LAVI), no significant change after bariatric surgery emerged from the meta-analysis of six studies (SMD, −0.01 ± 0.18, CI: −0.37/0.34, *p* = 0.95).

**Figure 4:**
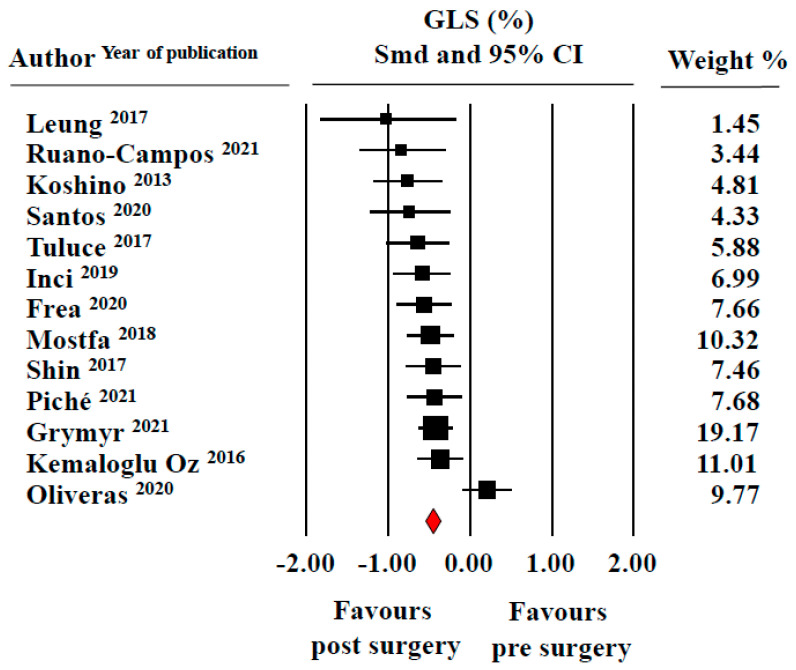
Forest plot for standard means difference (SMD) of left ventricular global longitudinal strain (LV GLS) in patients with obesity before and after bariatric surgery (random model). Relative weight of each study is reported on the right side. CI—confidence intervals. The filled squares represent the sample size of the study; the diamond is the average of the SMD.

Baseline and post-surgery mean LV GLS values in the pooled study population ranged from −11.3 ± 4.3% to −21.0 ± 2.3% and from −14.1 ± 3.9% to −26.5 ± 1.9%, the average pooled values being −16.9 ± 0.8% and −19.9 ± 1.0%, respectively. Figure 4 depicts the results of the meta-analysis where SMD suggested a significant improvement in LV mechanics after bariatric treatment (0.50 ± 0.08, CI 0.34/0.64, *p* < 0.0001).

### 3.4. Publication Bias

The presence of a single study effect was excluded from sensitivity analysis; a relevant publication bias was not present for studies reporting LV GLS before and after bariatric surgery. The difference pre- and post- bariatric treatment of this index of systolic function was still present after correction for publication bias (SMD, 0.38 ± 0.05,CI: 0.46/0.29, *p* < 0.0001) (Figure S1).

### 3.5. Correlation Analyses

A meta-regression analysis between changes in BMI and myocardial GLS before and after bariatric procedures aimed to assess the impact of such variables on this index of systolic function did not reveal a significant relationship between SMD in GLS and changes in BMI (0.01, *p* = 0.07).

### 3.6. Sensitivity Analyses

A sensitivity analysis restricted to 11 studies with follow-up ≥ 6 months and providing data on both LVEF and GLS confirmed that GLS (SMD: 0.47 ± 0.05, CI: 0.52/0.30, *p* < 0.0001) (Figure S2) but not LVEF (SMD: 0.14 ± 0.11, CI: −0.08/0.37, *p* = 0.21) improved after bariatric surgery (Figure S3).

## 4. Discussion

The influence of obesity on LV structure and function has been well-established. Overweight and obesity are associated with impaired LV mechanics, including a reduced GLS [30]. Unfortunately, data about the influence of bariatric surgery on LV function and mechanics in patients with morbid obesity are scarce, and our meta-analysis revealed several interesting, important findings that deserve to be commented on: (i) LVMI significantly decreased after bariatric surgery; (ii) LVEF, a conventional parameter of LV systolic function, did not change significantly after surgery; (iii) LV diastolic function, evaluated by mitral E/A and E/e’ ratios, improved after surgery compared to baseline; (iv) GLS was significantly better after bariatric surgery; (v) GLS emerged as a more sensitive parameter than LVEF in detecting LV systolic function improvement both in the main analysis and in that restricted to studies with follow-up ≥ 6 months.

The effect of bariatric surgery on body weight reduction can hardly be distinguished from the concomitant favourable effects of this procedure on comorbidities (hypertension, diabetes) frequently related to morbid obesity (16,18,24–28). This issue is clearly underlined by the evidence that in 7 out of 13 studies included in this meta-analysis, morbid obesity was associated with prevalent obesity and hypertension. This means that the mechanisms underlying the improvement in systolic function after bariatric surgery go beyond weight reduction and may vary in relation to comorbidities.

It should be pointed out, however, that BP and glucose decrements after bariatric surgery did not reach statistical significance in several studies or were even absent in others. In front of that, bariatric surgery significantly improved LV structural, functional and mechanical parameters in these patients [20]. Of note, the studies involving only patients with severe obesity without comorbidities showed a significant LV improvement [15]: this finding further underlines the importance of hemodynamic changes in obese patients who have undergone to bariatric surgery [31,32]. A meta-analysis including 110 patients followed for a median period of 9.7 months after bariatric surgery demonstrated that weight loss was related to significant reductions in heart rate, mean arterial pressure, resting oxygen consumption, as well as pulmonary capillary wedge pressure and mean pulmonary artery pressure [33]. Snelder et al. found a significant reduction of multiple biomarkers in patients followed for 12 months after bariatric surgery (72 out of 92 investigated biomarkers); in particular, the greatest improvement was reported for biomarkers related to insulin resistance and inflammation [32]. Recent findings also report a positive effect of bariatric surgery on the renin–angiotensin–aldosterone system (reduction in plasma renin activity, plasma aldosterone and angiotensin-converting enzyme 2 activity) associated with a reduction in LVMI and improvement in E/e’ [24].

Overall, these improvements may explain the reverse LV remodelling after bariatric surgery; previous meta-analyses summarized these improvements mainly focusing on LVMI reduction, LV geometry changes and improvement of LV diastolic function [12,13]. The influence of weight reduction after bariatric surgery on GLS improvement is difficult to separate from the decrease in LVMI, which is an important determinant of GLS.

Our meta-analysis documented that the LVEF did not change significantly after bariatric surgery. Some studies did not report any LVEF improvement [16,19,20,22,26]; this was not the case with GLS, which was significantly improved in all examined studies [16,17,18,19,20,21,22,23,24,25,26,27,28]. The present meta-analysis also demonstrated that only GLS, but not LVEF, was improved regardless of the duration of the follow-up. This underlines the higher sensitivity of GLS over LVEF in detecting subtle changes in LV systolic function. Some authors reported an improvement in LV circumferential strain in patients with morbid obesity after bariatric surgery, but in most studies, these changes did not reach statistical significance [16,19].

The parameters more frequently associated with GLS improvement were reduction in BMI and BP [16,22,26]. Our meta-analysis failed to demonstrate a relationship between BMI reduction and GLS improvement after bariatric surgery. Grymyr et al. suggested that change in LV geometry, particularly a reduction in eccentric LVH prevalence, one year after bariatric surgery may contribute to GLS improvement [26]. The influence of gender on GLS increase should be taken into account, as GLS improved more significantly in men than in women (6.7% vs. 3.9%, *p* < 0.05) [26].

Several important clinical implications of the current meta-analysis should be considered. Due to the overall pandemic of obesity, the number of patients undergoing bariatric surgery will likely increase: it is, therefore, of importance to predict the level of reverse LV remodelling and to identify the more sensitive parameters in detecting these improvements. GLS has become the standard of care in a wide spectrum of cardiovascular disorders not only because of its better sensitivity and reproducibility but even more of its better predictive value over conventional LVEF [34]. Although the predictive value of GLS in patients with obesity after bariatric surgery is supported by a limited number of studies, existing data in the setting of bariatric surgery strongly support the view that GLS should be evaluated at baseline and during follow-up.

## 5. Limitations

Several limitations of the current meta-analysis need to be mentioned. A first limitation is the clinical heterogeneity, such as differences in age, sample size, sex, BMI, comorbidities, type of surgical procedure and duration of follow-up between the various studies. This was also the case for statistical heterogeneity. Second, the high risk of bias present in studies without a control group (comparability domain) may have influenced the results of the meta-analysis. GLS assessment is highly dependent on the quality of echocardiographic images, although reproducibility is up to 95%. Of note, the significant reduction in body weight may have partly contributed to improving GLS assessment during follow-up due to the better quality of images. Moreover, in the majority of included studies, a GE echocardiographic machine was used, and GLS was evaluated by EchoPac; only a minority of studies used a Philips echocardiographic machine for examination and TomTec for strain analysis. Finally, it should be pointed out that the inter-vendor difference reported for GLS assessment is quite limited and acceptable [35].

## 6. Conclusions

The present meta-analysis showed a significant improvement in LV structure and diastolic function as well as cardiac mechanics after bariatric surgery. In particular GLS was proven to be a more sensitive parameter in assessing changes in LV systolic function following the bariatric procedure than LVEF. From a clinical perspective, a comprehensive evaluation of the effects of bariatric surgery should include the assessment of cardiac mechanics. However, further studies are needed to investigate whether changes in cardiac mechanics induced by bariatric surgery correlate better with cardiovascular prognosis than conventional echocardiographic parameters.

## Figures and Tables

**Table 1:** Summary of 13 studies targeting left ventricular strain in patients before and after bariatric surgery, as assessed by echocardiography, published from 2013 to 2021.

Author [Reference] Publication Year	Sample Size (n)	Age (Years)	Sex (% Male)	BMI Pre (kg/mq)	BMI Post Surgery (kg/mq)	GLS Pre (%)	GLS Post Surgery (%)	Type of Surgery	Mean Follow-Up (Months)	STE Method	Comorbidities
**Koshino** [16] 2013	28	52 ± 12	29	51 ± 9	37 ± 10	−11.3 ± 4.3	−14.1 ± 3.9	Gastric banding, biliopancreatic diversion with duodenal switch or Roux-en-Y surgery	23	2D	Prevalent hypertension and type 2 diabetes
**Kemaloglu Oz** [17] 2016	53	37 ± 11	38	49.1 ± 8	36.9 ± 6.0	−21 ± 2.3	−26 ± 3	Sleeve gastrectomy	6	2D	None
**Leung** [18] 2017	10	na	na	44.3 ± na	34.5 ± na	−13 ± na	−19.3 ± na	Sleeve gastrectomy	12	2D	Type 2 diabetes
**Shin** [19] 2017	37	36 ± 10	30	39.7 ± 6	27.9 ± 4.0	−14.1 ± 1.9	−16.2 ± 1.4	Sleeve gastrectomy	15.6	2D	None
**Tuluce** [20] 2017	32	34 ± 9	22	44 ± 4	38.9 ± 4	−14.5 ± 3.2	−15.9 ± 2.8	Sleeve gastrectomy	1	2D	None
**Mostfa** [21] 2018	52	38 ± 6	35	42.3 ± 3	28.5 ± na	−17.2 ± 2.1	−22,7 ± 3.9	Gastric bending	6	2D	None
**Inci** [22] 2019	37	na	27	44.1 ± 3	33.5 ± na	−16.1 ± 1.3	−17.5 ± 1.1	Sleeve gastrectomy	6	2D	None
**Frea** [23] 2020	40	42 ± 11	28	44 ± 5	31 ± 5	−17 ± 2	−20 ± 1	Sleeve gastrectomy or Roux-en-Y surgery	10	2D	Prevalent LVH
**Oliveras** [24] 2020	45	44 ± 9	24	42.5 ± 6	29.8 ± na	−19.1 ± 2.8	−18.4 ± na	Sleeve gastrectomy or Roux-en-Y surgery	12	2D	Prevalent hypertension and OSA
**Santos** [25] 2020	25	35 ± 8	6	46.8 ± 6	38.4 ± 5.0	−17.4 ± 3.2	−19.2 ± 2.7	Sleeve gastrectomy	3.6	2D	Prevalent hypertension
**Grymyr** [26] 2021	94	43 ± 10	29	41.8 ± 5	28.5 ± 5.0	−15.8 ± 4.8	−20.4 ± 2.8	Roux-en-Y surgery	12	2D	Prevalent hypertension and type 2 diabetes
**Piché** [27] 2021	38	42 ± 11	11	48.4 ± 7	35.4 ± 6.0	−16.3 ± 2.5	−18.2 ± 1.9	Biliopancreatic diversion with duodenal switch	6	2D	Prevalent hypertension, type 2 diabetes and OSA
**Ruano-Campos** [28] 2021	21	47 ± 2	33	46.8 ± 1	29.6 ± 1.0	−19.8 ± 0.5	−22.2 ± 0.4	Single anastomosis duodeno-ileal bypass with sleeve gastrectomy	9.2	2D	Prevalent hypertension and type 2 diabetes

BMI—body mass index; GLS—global longitudinal strain; LVH—left ventricular hypertrophy; OSA—obstructive sleep apnea; STE—speckle tracking echocardiography. Data are presented as absolute numbers, percentage and mean ± SD.

## Data Availability

Data can be provided upon reasonable request.

## Supplementary Materials

The following supporting information can be downloaded at: https://www.mdpi.com/article/10.3390/jcm11164655/s1. Figure S1. Funnel plot assessing publication bias for standard means difference (SMD) of LV GLS in patients with obesity before and after bariatric surgery. Observed (white symbols) and imputed (black symbols) values. Figure S2. Forest plot for standard means difference (SMD) of LV GLS in patients with obesity before and after bariatric surgery in studies with follow-up duration >6 months (random model). Relative weight of each study is reported on the right side. CI—confidence intervals. The filled squares represent the sample size of the study; the diamond is the average of the SMD. Figure S3. Forest plot for standard means difference (SMD) of LVEF in patients with obesity before and after bariatric surgery in studies with follow-up duration >6 months (random model). Relative weight of each study is reported on the right side. CI—confidence intervals. The filled squares represent the sample size of the study; the diamond is the average of the SMD. Table S1: Search Strategy. Table S2: Newcastle-Ottawa scale quality assessment form for the studies included in the review.
