# Supplementary material for: Bariatric Surgery and Myocardial Mechanics: A Meta-Analysis of Speckle Tracking Echocardiographic Studies
Source: J Clin Med. 2022 Aug 9;11(16):4655. doi: 10.3390/jcm11164655 (PMC9410478; doi:10.3390/jcm11164655)
Supplement: Supplementary file 1 [file jcm-11-04655-s001.zip › jcm-1829359-supplementary.pdf]

Table S1 Search Strategy

Source	Search Strategies
**Pubmed**	#1 BARIATRIC SURGERY<br>n=39285<br>#1 and OBESITY, LEFT VENTRICULAR MECHANICS, LEFT VENTRICULAR HYPERTROPHY, SYSTOLIC DYSFUNCTION, GLOBAL LONGITUDINAL STRAIN, ECHOCARDIOGRAPHY, STE ECHOCARDIOGRAPHY.<br>n=1208<br>Last Search date:2022/05/31
**OVID MEDLINE**	#1 BARIATRIC SURGERY<br>n=8347<br>#1 and OBESITY, LEFT VENTRICULAR MECHANICS, LEFT VENTRICULAR HYPERTROPHY, SYSTOLIC DYSFUNCTION, GLOBAL LONGITUDINAL STRAIN, ECHOCARDIOGRAPHY, STE ECHOCARDIOGRAPHY<br>n=436<br>Last Search date:2022/05/31
**The Cochrane library**	#1 BARIATRIC SURGERY<br>n=76<br>#1 and OBESITY, LEFT VENTRICULAR MECHANICS, LEFT VENTRICULAR HYPERTROPHY, SYSTOLIC DYSFUNCTION, GLOBAL LONGITUDINAL STRAIN, ECHOCARDIOGRAPHY, STE ECHOCARDIOGRAPHY.<br>n=0<br>Last Search date:2022/05/31

Table S2. Newcastle-Ottawa scale quality assessment form for the studies included in the review.

Study & Year	Selection				Comparability	Outcome			Quality Score
	1	2	3	4	5	6	7	8	(*)
**Koshino**[16] 2013	1	1	1	1	2	1	1	1	**9**
**Kemaloglu Oz**[17] 2016	1	0	1	1	2	1	1	1	**8**
**Leung**[18] 2017	0	1	1	1	2	1	1	1	**8**
**Shin**[19] 2017	1	0	1	1	0	1	1	1	**6**
**Tuluce**[20] 2017	1	1	1	1	2	1	0	1	**8**
**Mostfa**[21] 2018	1	0	1	1	0	1	1	1	**6**
**Inci**[22] 2019	1	0	1	1	0	1	1	1	**6**
**Frea**[23] 2020	1	0	1	1	0	1	1	1	**6**
**Oliveras** [24] 2020	1	0	1	1	0	1	1	1	**6**
**Santos**[25] 2020	1	0	1	1	0	1	1	1	**6**
**Grymyr** [26] 2021	1	0	1	1	0	1	1	1	**6**
**Piché**[27] 2021	1	1	1	1	2	1	1	1	**9**
**Ruano-Campos**[28] 2021	1	0	1	1	0	1	1	1	**6**

1)Representativeness of the exposed cohort (1 point) ; 2) Selection of the non-exposed cohort; 3) Ascertainment of exposure (1 point); 4) Demonstration that outcome of interest was not present at start of study ; 5) Comparability of cohorts on the basis of the design or analysis controlled for confounders (2 point); 6) Assessment of outcome (1 point); 7) Was follow-up long enough for outcomes to occur (1 point) ; 8) Adequacy of follow-up of cohorts (1 point).

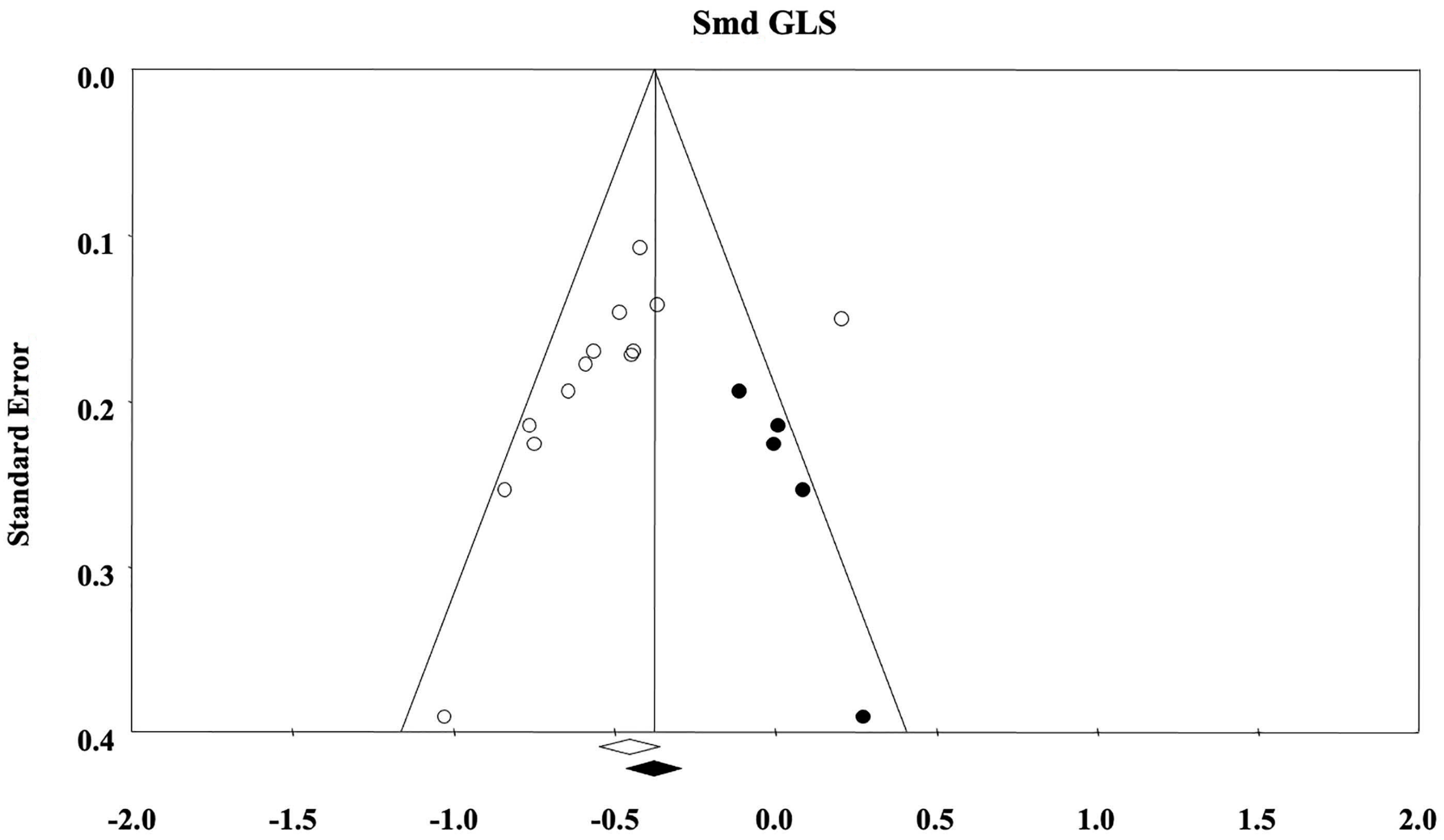

Figure S1. Funnel plot assessing publication bias for standard means difference (SMD) of LV GLS in patients with obesity before and after bariatric surgery. Observed (white symbols) and imputed (black symbols) values.

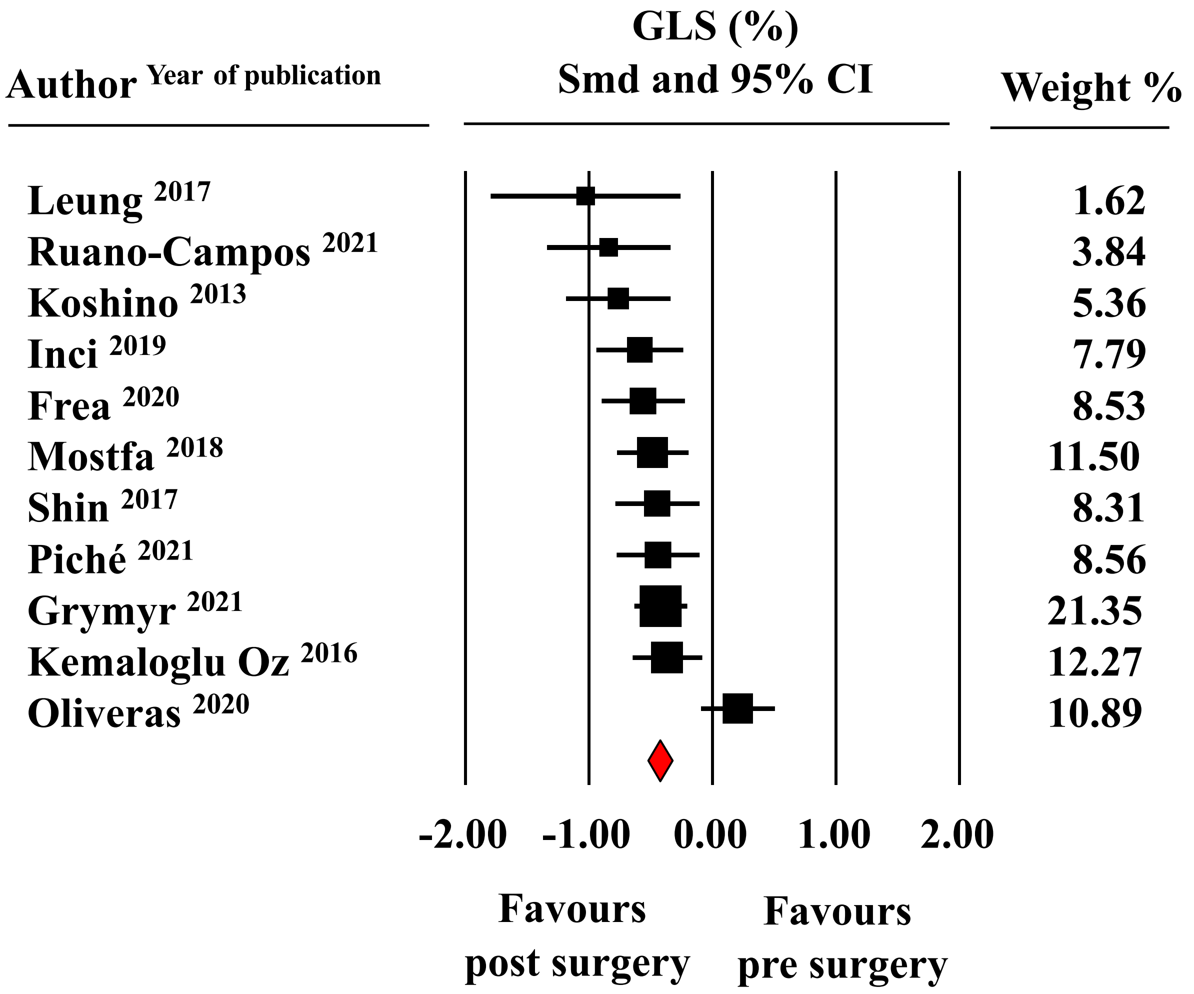

Figure S2. Forest plot for standard means difference (SMD) of LV GLS in patients with obesity before and after bariatric surgery in studies with follow-up duration >6 months (random model). Relative weight of each study is reported on the right side. CI—confidence intervals. The filled squares represent the sample size of the study; the diamond is the average of the SMD.

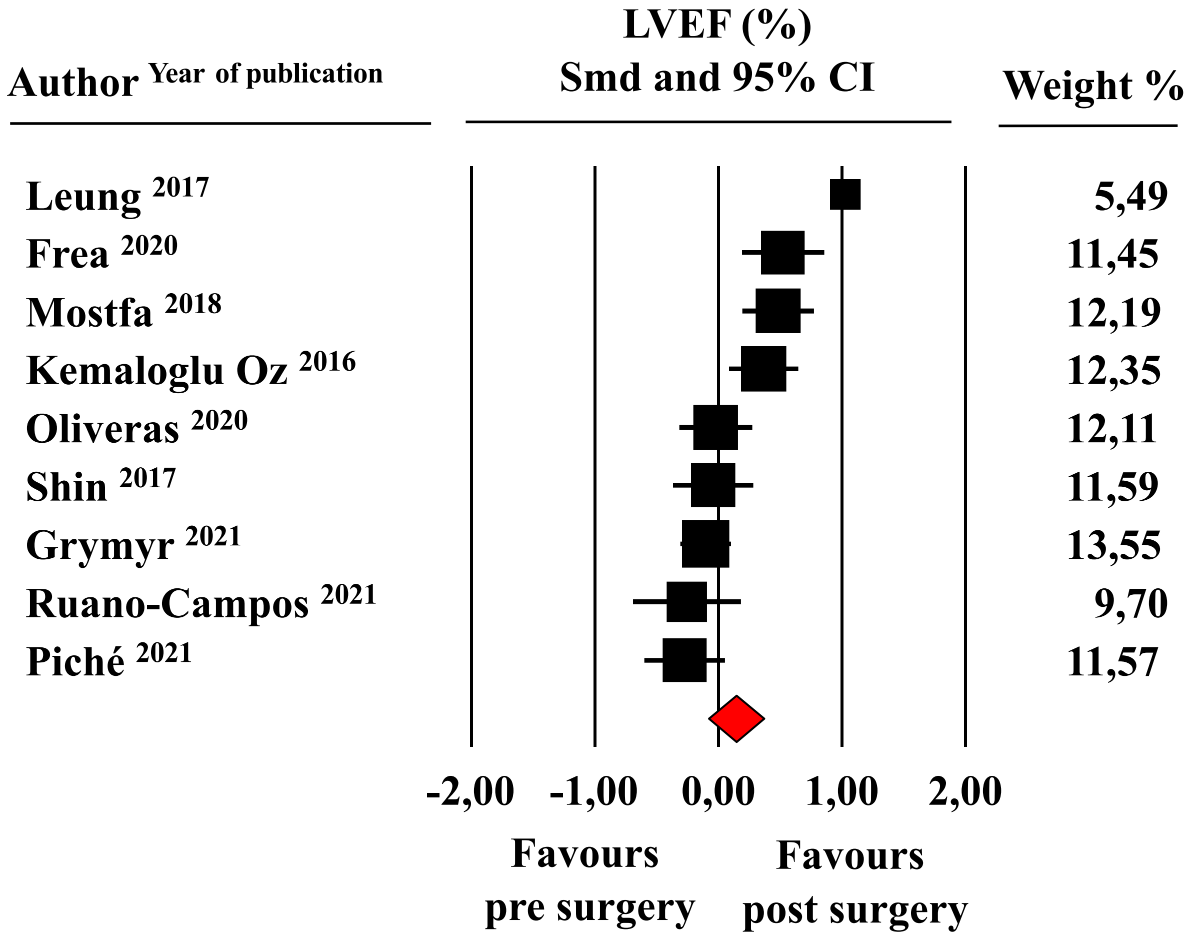

Figure S3. Forest plot for standard means difference (SMD) of LVEF in patients with obesity before and after bariatric surgery in studies with follow-up duration >6 months (random model). Relative weight of each study is reported on the right side. CI—confidence intervals. The filled squares represent the sample size of the study; the diamond is the average of the SMD.
